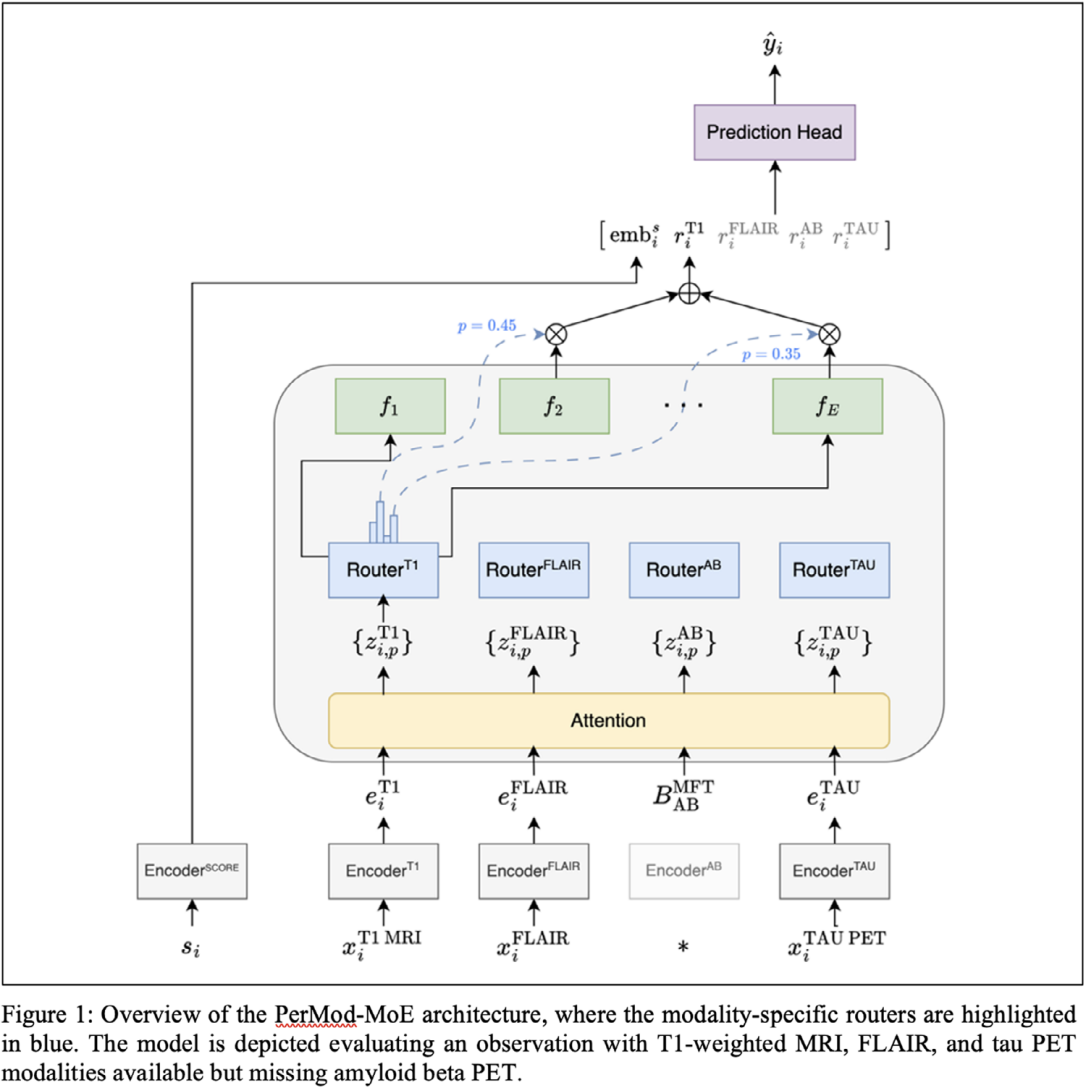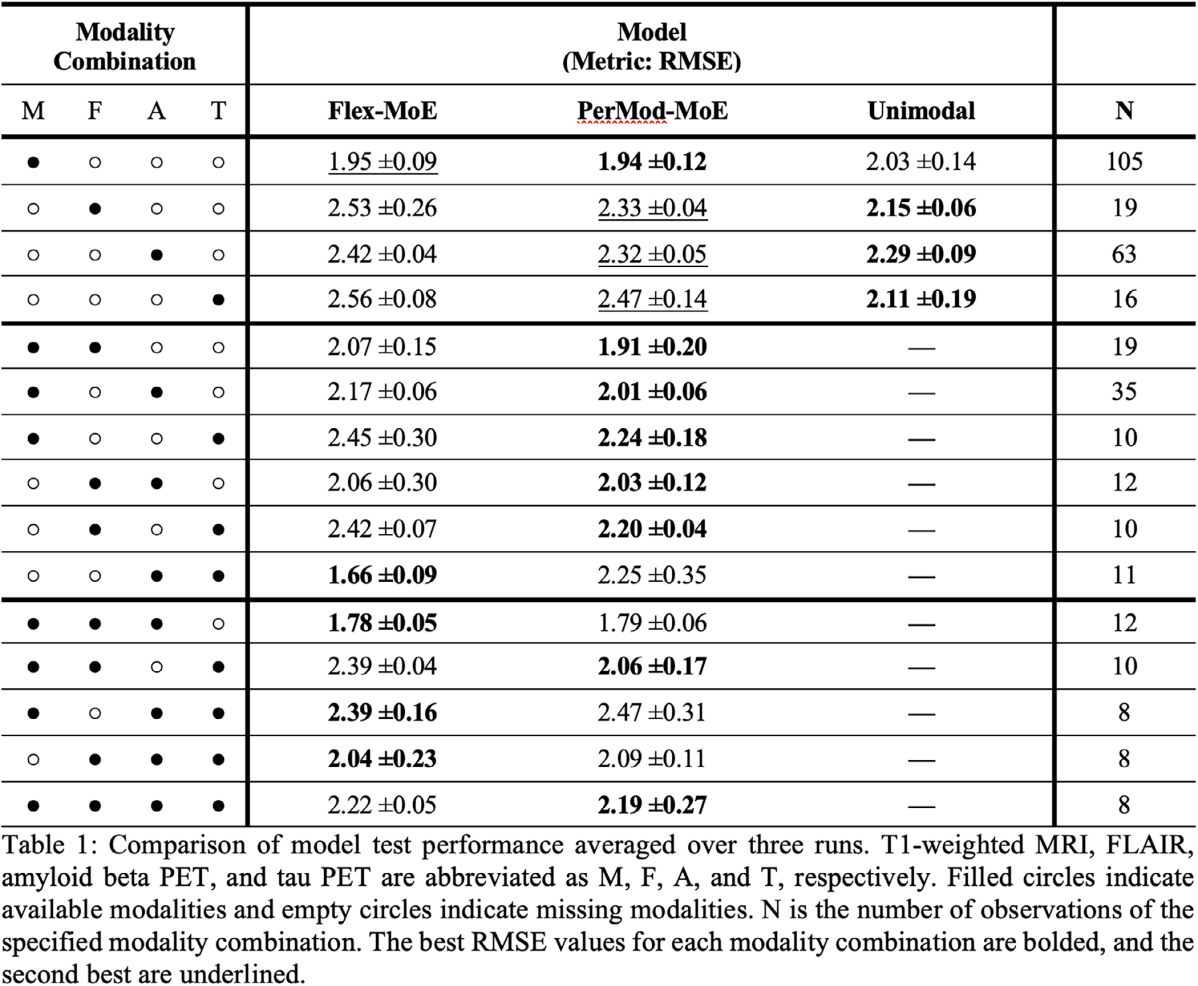# Flexible Multimodal Fusion of Neuroimaging Modalities for Alzheimer's Disease Progression Prediction

**DOI:** 10.1002/alz70861_108442

**Published:** 2025-12-23

**Authors:** Benjamin Burns, Yuan Xue, Douglas W. Scharre, Xia Ning

**Affiliations:** ^1^ The Ohio State University, Columbus, OH USA; ^2^ Ohio State University Wexner Medical Center, Columbus, OH USA

## Abstract

**Background:**

Alzheimer’s disease (AD) progression has a high inter‐patient variance, with some patients slowly progressing from mild cognitive impairment (MCI) to dementia while others demonstrate more rapid cognitive decline. AD progression prediction with deep learning is enhanced by incorporating multiple neuroimaging modalities, but existing multimodal models are limited by severe predictive performance degradation when some modalities are missing during inference.

**Method:**

T1‐weighted MRI, FLAIR, amyloid beta PET, and tau PET neuroimaging data were obtained for 469 patients with MCI from the Alzheimer’s Disease Neuroimaging Initiative (ADNI) dataset. The state of the art in flexible multimodal fusion, Flex‐MoE, was modified by replacing the sparse mixture‐of‐experts router with independent routers for each neuroimaging modality. The resulting method is referred to as **Per**‐**Mod**ality **M**ixture‐**o**f‐**E**xperts (PerMod‐MoE). Flex‐MoE and PerMod‐MoE were evaluated along with unimodal neuroimaging models on predicting two‐year change in Clinical Dementia Rating‐Sum of Boxes (CDR‐SB).

**Result:**

PerMod‐MoE achieves 8%, 4%, and 4% improvements in root mean square error (RMSE) over Flex‐MoE when evaluated on observations with only FLAIR, amyloid beta PET, and tau PET, respectively. Further, PerMod‐MoE demonstrates competitive performance with Flex‐MoE when more modalities are available. PerMod‐MoE boasts an average 13% improvement in RMSE for patients with an initial CDR‐SB greater than five and an average 16% improvement for patients with an observed two‐year CDR‐SB increase of 0.5 or 1.

**Conclusion:**

PerMod‐MoE, with its addition of independent, modality‐specific routers in the sparse mixture‐of‐experts layer, improves performance on AD progression prediction when neuroimaging modality availability is severely limited during inference. PerMod‐MoE offers a novel approach to effectively leverage available neuroimaging data for more accurate AD progression prediction by clinical providers.